# Anterior cervical corpectomy and fusion with stand-alone cages in patients with multilevel degenerative cervical spine disease is safe

**DOI:** 10.1186/s12891-021-04883-5

**Published:** 2022-01-03

**Authors:** Mohamed H. Tohamy, Georg Osterhoff, Ahmed Shawky Abdelgawaad, Ali Ezzati, Christoph-E. Heyde

**Affiliations:** 1 Spine Unit, Martin-Ulbrich-Haus Rothenburg, Horkaer Str. 15-21, 02929 Rothenburg, Oberlausitz Germany; 2 Spine Departement, Helios Klinikum Erfurt, Nordhäuser Str. 74, 99089 Erfurt, Germany; 3 Ligamenta Spine Center, Walter-Kolb-Street 9-11, 60594 Frankfurt am Main, Germany; 4 Department of Orthopedics, Trauma and Plastic Surgery, University Hospital Leipzig, Liebigstrasse 20, 04179 Leipzig, Germany; 5 Department of Orthopedic and Trauma Surgery, Assiut University Hospitals, Assiut, Egypt; 6 Department of Orthopedics, Trauma and Plastic Surgery, University Hospital Leipzig, 04103 Leipzig, Germany

**Keywords:** Cervical spine, Stand-alone cage, Corpectomy, Spinal canal stenosis, Anterior plate, Anterior cervical decompression, Cervical fusion, Dysphagia

## Abstract

**Background:**

In case of spinal cord compression behind the vertebral body, anterior cervical corpectomy and fusion (ACCF) proves to be a more feasible approach than cervical discectomy. The next step was the placement of an expandable titanium interbody in order to restore the vertebral height. The need for additional anterior plating with ACCF has been debatable and such technique has been evaluated by very few studies. The objective of the study is to evaluate radiographic and clinical outcomes in patients with multilevel degenerative cervical spine disease treated by stand-alone cages for anterior cervical corpectomy and fusion (ACCF).

**Methods:**

Thirty-one patients (66.5 ± 9.75 years, range 53–85 years) were analyzed. Visual Analog Scale (VAS) and the 10-item Neck Disability Index (NDI) were assessed preoperatively and during follow-up on a regular basis after surgery and after one year at least. Assessment of radiographic fusion, subsidence, and lordosis measurement of Global cervical lordosis (GCL); fusion site lordosis (FSL); the anterior interbody space height (ant. DSH); the posterior interbody space height (post. DSH); the distance of the cage to the posterior wall of the vertebral body (CD) were done retrospectively. Mean clinical and radiographic follow-up was 20.0 ± 4.39 months.

**Results:**

VAS-neck (*p* = 0.001) and VAS-arm (*p* < 0.001) improved from preoperatively to postoperatively. The NDI improved at the final follow-up (p < 0.001). Neither significant subsidence of the cages nor significant loss of lordotic correction were seen. All patients showed a radiographic union of the surgically addressed segments at the last follow up.

**Conclusions:**

Application of a stand-alone expandable cage in the cervical spine after one or two-level ACCF without additional posterior fixation or anterior plating is a safe procedure that results in fusion. Neither significant subsidence of the cages nor significant loss of lordotic correction were seen.

**Trial registration:**

Retrospectively registered. According to the Decision of the ethics committee, Jena on 25th of July 2018, that this study doesn’t need any registration. https://www.laek-thueringen.de/aerzte/ethikkommission/registrierung/.

**Supplementary Information:**

The online version contains supplementary material available at 10.1186/s12891-021-04883-5.

## Background

Cervical spine fusion is an international set process for handling degenerative cervical spine problem [1, 2].

Anterior cervical discectomy and fusion (ACDF) is used in treating symptomatic cervical spondylosis, degenerative disc disease and spinal canal stenosis (SCS), which results in a compression of one or more nerve roots and/or the spinal cord. The suitability and effectiveness of the technique have been proved in achieving functional recovery and easing the spinal and nerve pain. In addition, ACDF maintains the spinal column stability and promotes the sagittal balance [3–5]. In the case of foraminal stenosis, an anterior approach has the advantage that the disc as major contributor to nerve compression can be removed. In addition, the foramina can be addressed directly without weakening the posterior tension band.

For multilevel stenosis, however, decompression of the spinal canal may be insufficient through the disc space alone. Laminectomy and laminoplasty have proven their efficiency in these cases [6], but complications as C5 nerve root palsy, [7], axial neck pain, segmental instability and progressive cervical kyphosis are not infrequent [8, 9].

Alternatively, the application of expandable cages after Anterior Cervical Corpectomy and Fusion (ACCF), can prevent the damages in the posterior approach. ACCF from C3—C7 is being used to decompress and reconstruct the cervical spine for a wide variety of degenerative disorders, trauma, neoplasms and infectious disorders.

In degenerative diseases, the indication for corpectomy is the compression that extends beyond the disc space and behind the vertebral body (VB). In such cases, performing corpectomy is essential to achieve adequate spinal cord decompression. Followed by reconstruction, ACCF permits the most direct and proper decompression of the anterior spinal cord or resection of lesions that involve the cervical VBs and improve the cervical lordosis [10]. The technique minimally disrupts healthy cervical muscles and is associated with a low risk of injuring surrounding structures [11, 12]. It has been shown that an only-anterior approach for multiple levels can lead to good results in patients with ossification of the posterior longitudinal ligament (OPLL) and patients with a spinal canal occupancy ratio of ≥50% managed by anterior surgery alone had better results [13]. Even though dysphagia and dyspnea are potential complications, the anterior approach is associated with little morbidity and usually results in earlier postoperative recovery compared to open posterior procedures [14, 15]. Some surgeons, however, favour a combined anterior and posterior approach for multilevel fusion as anterior-alone constructs may be susceptible to failure and loss of correction because of mechanical disadvantages [16, 17]. Others suggested to add anterior plating in ACCF constructs in order to maintain sufficient stability until osseous fusion is achieved.

Hence, the aim of this study is to evaluate radiographic and functional outcome in a series of patients with multilevel degenerative cervical spine disease treated by stand-alone cages for ACCF.

## Methods

### Patients

All patients who had undergone one-or two level ACCF for SCS with or without cervical spondylosis affecting the levels C3/4 to C7/T1 in a tertiary spine center between 2014 and 2016 were identified in a retrospective chart review.

Further inclusion criteria were neck or radicular upper extremity pain and/or neurological deficit due to compression of nerve roots or the spinal cord confirmed by computed tomography (CT) and/or magnetic resonance imaging (MRI). Patients with previous surgery at the index level, systemic or local infection, rheumatoid arthritis (RA), non-controlled diabetes mellitus (DM), patients with a known allergy to any of the materials contained in the cage implant and patient with progressive malignancy within the last five years were excluded. None of the patients received osteo-anabolic therapy like teriparatide or denosumab.

In total, 31 patients (mean age 66.5 ± 9.75 years, range 53.0–85.00 years, 10 female) were included in this study (Table 1). One patient had OPLL, and six patients presented with neurological impairment without history of trauma which illustrated in (Suppl1).

**Table 1:** Patients’ baseline characteristics

	Number “*n* = 31“	Percent
Age		
< 60	10	32.3
60–70	8	25.8
70+	13	41.9
Range	53.0–85.00	
Mean ± S.D	66.5 ± 9.75	
Sex		
Male	21	67.7
Female	10	32.3
Level		
3	2	6.45
4	4	12.90
5	4	12.90
6	7	22.58
7	1	3.23
4 + 5	6	19.35
5 + 6	7	22.58

### Surgical technique

In all patients, ACCF was performed through a standard anterior cervical approach by microscopy-assisted dissection [18]. After a corpectomy and decompression, a cervical expandable titanium cage (X-Core® Mini, NuVasive, San Diego, USA) was introduced into the multilevel void. The cage was then expanded under fluoroscopic control until sufficient correction of the cervical spinal alignment could be achieved.

### Clinical evaluation

Clinical examinations were performed before surgery and at final follow-up. As a standard, this included documentation of neck and arm pain by the visual analog scale (VAS) [19] and assessment of functional outcome by the 10-item NDI (German version) [20]. Patient self-reported dysphagia-related symptoms were graded as “none,” “mild,” “moderate” and “severe” as previously described by Bazaz et al. [18]. Additionally, the amount of dysphagia-associated pain (VAS 0–10) and the duration of dysphagia-related symptoms were recorded.

### Radiologic assessment

Plain radiographs with an anteroposterior view and lateral views in flexion and extension were taken preoperatively, postoperatively and at final follow-up and were evaluated by three experienced spine surgeons.

Evaluation of global cervical lordosis (GCL) and fusion site lordosis (FSL):

Changes of the lordotic cervical alignment were measured using a modification of the method described by Faldini et al. of the cervical interbody fusion [21].

**Fig. 1:**
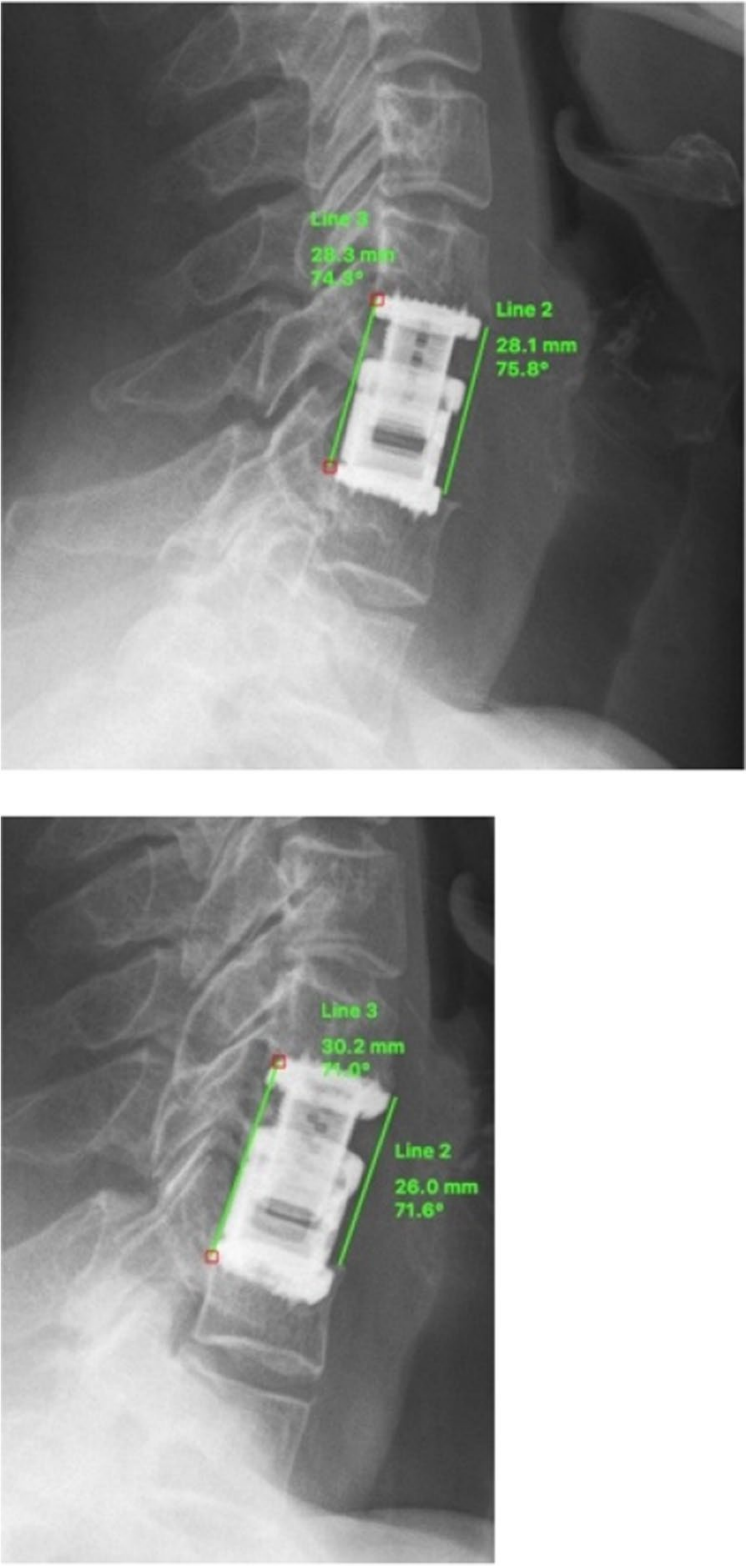
Measurement of Fusion Site Lordosis (FSL) postoperatively (**left**), then I would like to put the pic. on the left side or we have to write (up) and at last follow-up (**right**), then I would like to put it on the right side or we have to write down instead of right

GCL was measured between the upper endplate of C3 and the lower border of C7. FSL was measured between the upper endplate of the cranial fusion site vertebra and the lower endplate of the caudal fusion site vertebra (bi-segmental FSL, Fig. 1). For cervical alignment, positive values represent lordosis while negative values indicate kyphotic alignment.

### Evaluation of cage migration and subsidence

**Fig. 2:**
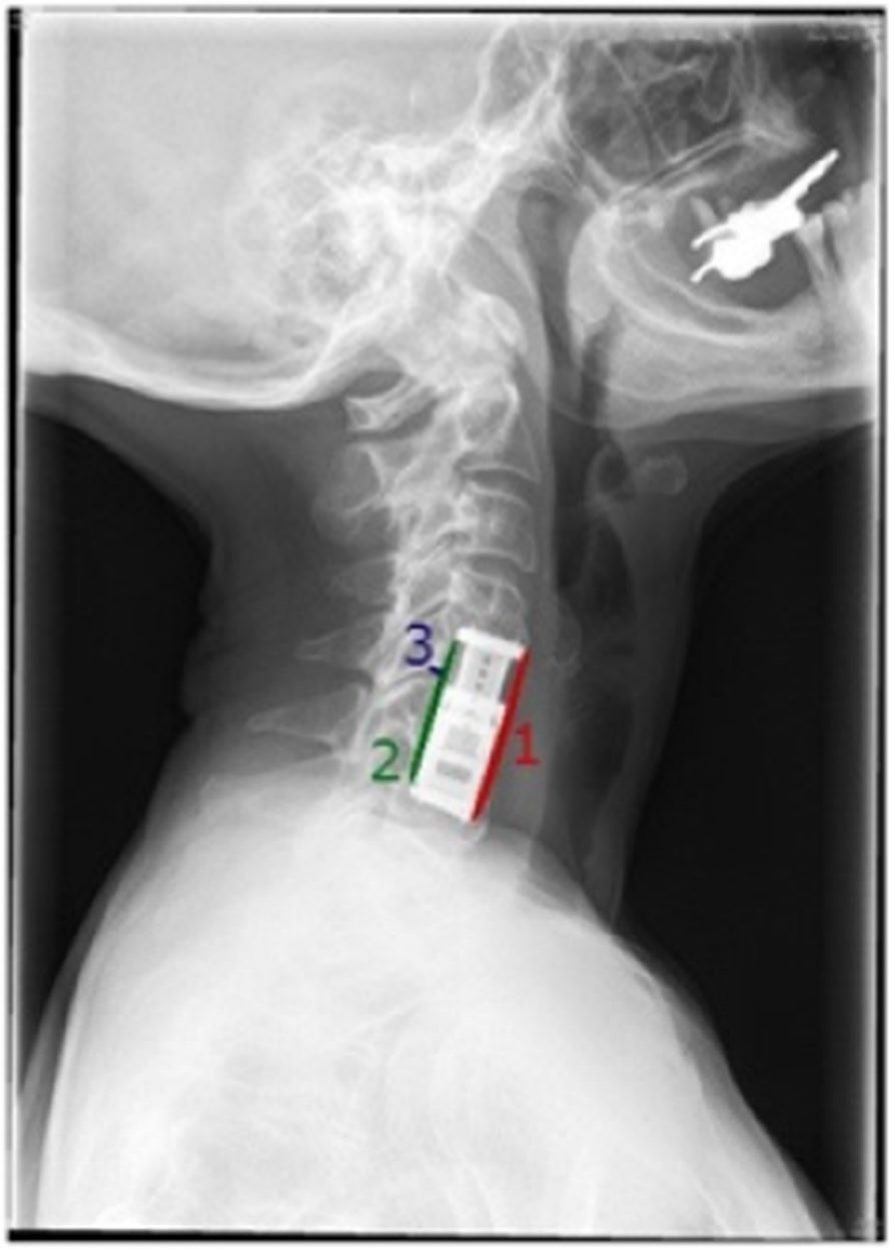
Measurement of cage migration and subsidence1: anterior DSH, 2: posterior DSH, 3: CD. Modified Gercek et al.

The same method as described by Gercek et al. was used [22]. Cage migration and subsidence were assessed by measuring the distance between the posterior edge of the implant and the posterior wall of the lower endplate as well as the anterior and posterior interbody space height on lateral plain radiographs postoperatively and at the end of the follow-up. Migration or settling were defined as changes of 3 mm or more of the following parameters: the anterior interbody space height (ant. DSH), the posterior interbody space height (post. DSH) and the distance of the cage to the posterior wall of the vertebral body (CD) [22] (Fig. 2).

### Evaluation of the fusion

Fusion was evaluated as decribed by (Choudhri, T. F., Mummaneni) [23], (Lee CS, Chung SS, Choi SW, et al.) [24] and (Cannada LK, Scherping SC, Yoo JU, et al.) [25].

**Fig. 3:**
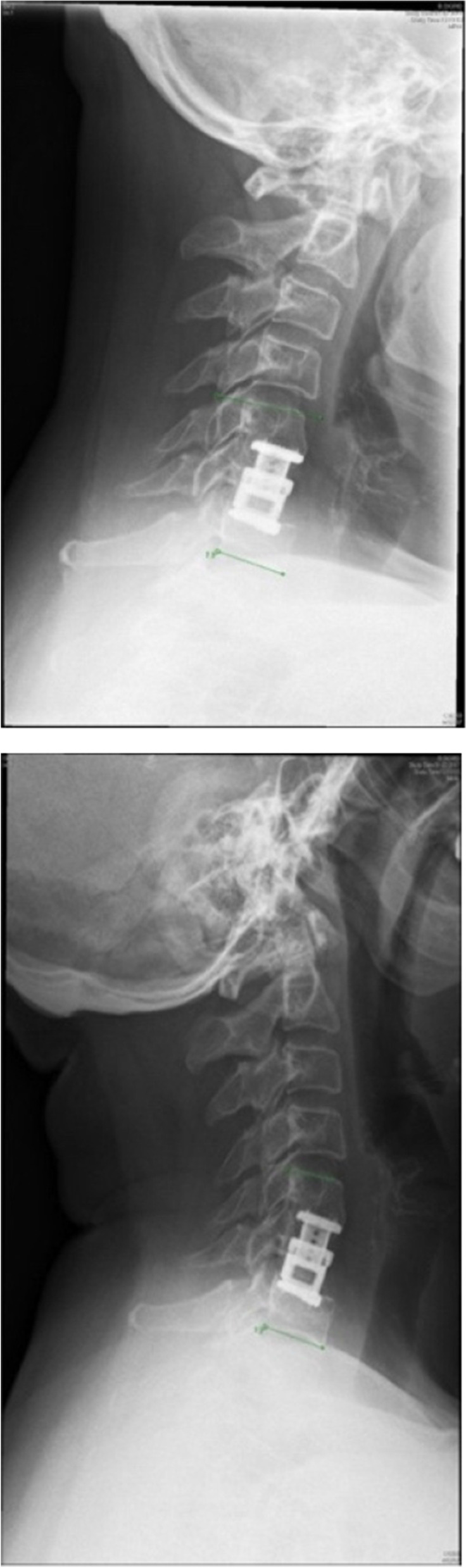
Evaluation of fusion: Changes in flexion and extension of the lateral view of the cervical spine of more than 2 mm indicate nonunion

**Fig. 4:**
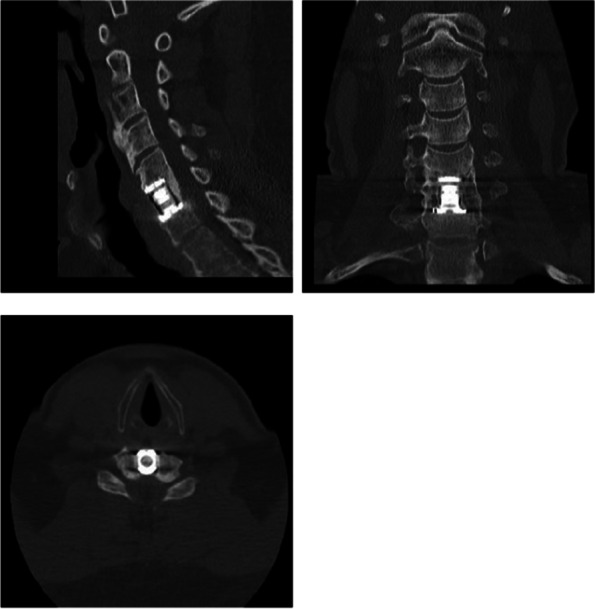
A CT scan at final follow-up shows fusion with presence of bone mass inside the cage

**Fig. 5:**
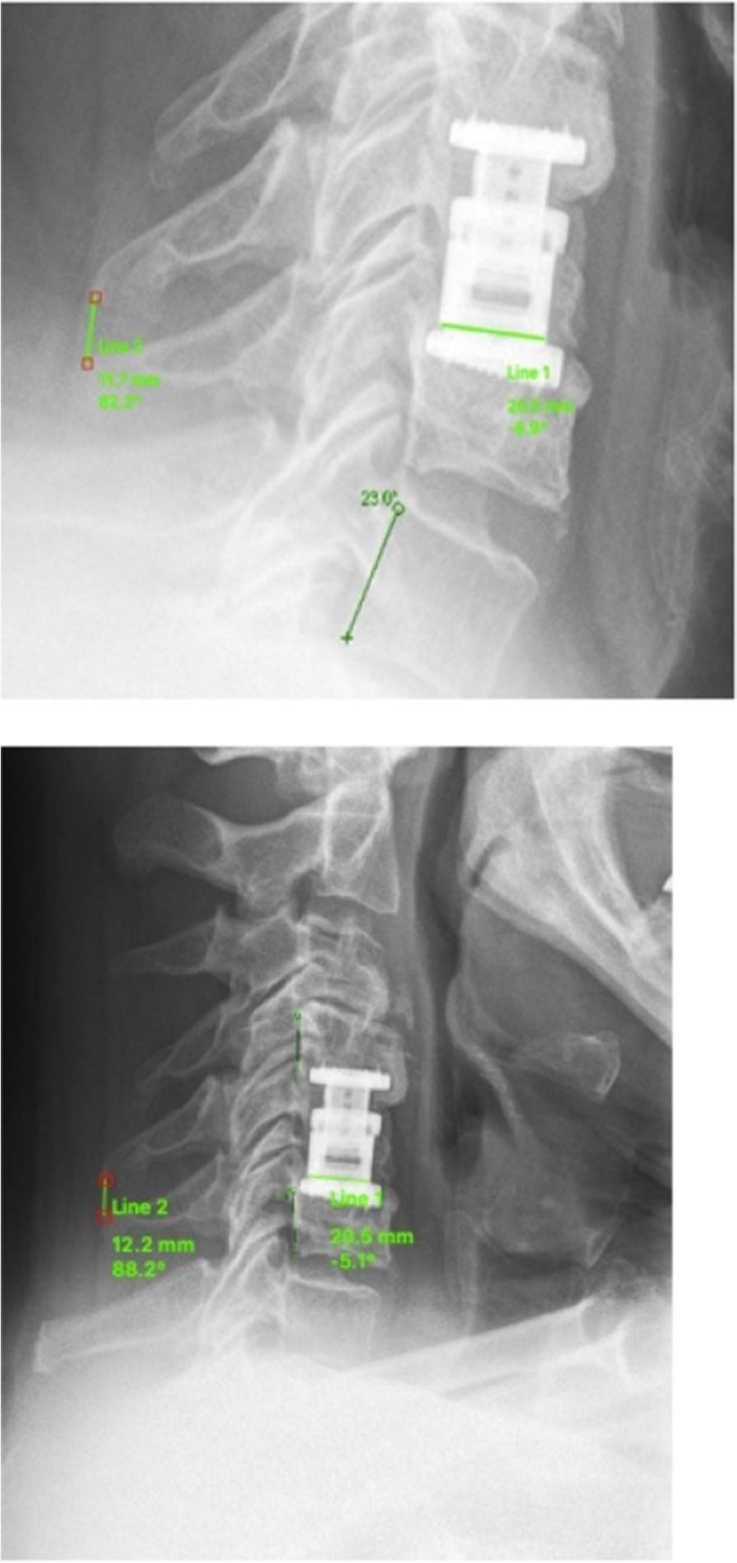
Distance between the tips of the spinous processes on flexion is 12.2 mm and 11.7 mm in extension in lateral views

The first criterion indicating nonunion was change in endplate angles in flexion and extension in the lateral view of more than 2 degrees (Fig. 3). The second criterion for nonunion was a change of more than 2 mm in the distance between the tips of the spinous processes of the surgically managed levels on flexion and extension lateral views (Fig. 4 and Fig. 5) [25]. In case one of these two criteria was noticed, the segment was considered not fused.

**Fig. 6:**
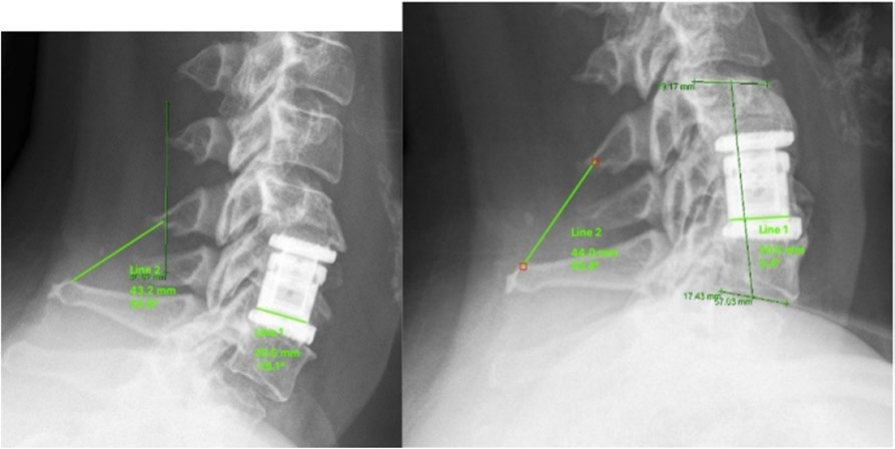
Distance between the tips of the spinous processes on flexion is 44 mm and 43.2 mm in extension of lateral views

If continuous trabecular bone bridges could be confirmed at 12 months FU (at any one of the following locations: anterior, posterior, lateral or within the cage), the segment was classified as fused. In case of absence of any bony bridge or confirmation of a complete bony discontinuity within the disc space, the segment was classified as not fused [26] as illustrated in (Fig. 6).

In case of potential nonunion in the radiographic evaluation, CT (sagittal and coronal two-dimensional) was used.

The height as well as the angle of the fused segment C2–C7 were measured and analyzed preoperatively immediately after surgery and at the final follow-up.

Mean clinical and radiographic follow-up was 20.0 ± 4.39 months (range 12 to 27 months).

### Statistical analysis

Statistical analysis was done using Statistical Package for Social Sciences (SPSS/version 20, SPSS Inc., Chicago, IL, USA) [27].

Unless otherwise denoted, data was summarized as mean ± standard deviation.

Student’s t-test was used to compare differences in means between two groups and an.

ANOVA test was used for parametric data to distinguish between three groups. Spearman-Rho coefficients were calculated to assess potential bivariate associations. The level of significance was defined as *p* < 0.05.

## Results

### Clinical outcome

The preoperative VAS-neck increased postoperatively (*p* = 0.013) and then improved at final follow-up (*p* = 0.001). The preoperative VAS-arm decreased (p = 0.001) at the end of follow up, the VAS-arm was not statistically different to the postoperative (*p* = 0.336).

The value for the NDI improved from an initial presentation (p = 0.001) and at final follow-up (p = 0.001). No revision surgeries were necessary.

As for dysphagia-related symptoms, 20 patients out of 31 patients (64.5%) had mild symptoms like difficulty in swallowing. In all cases, these symptoms disappeared within two days after surgery. No patient had dysphagia at the time of discharge.

### Radiographic outcome

Evaluation of global cervical lordosis (GCL) and fusion site lordosis (FSL):

Compared to the preoperative measurement (11.8° ± 8.19°), GCL was significantly increased postoperatively (15.8° ± 7.34°, *p* = 0.025) and at final follow up 14.9° ± 7.12°, *p* = 0.021) while these postoperative measurements showed an insignificant difference (*p* = 0.319). In contrast, postoperative (7.5° ± 3.90°) and follow-up FSL measurements (7.9° ± 5.41°) showed no significant differences compared to the preoperative values (7.1° ± 4.35°, *p* = 0.261/0.379).

### Cage migration and subsidence

The comparison between anterior DSH, posterior DSH and the CD postoperatively and at last follow-up showed no change in anterior and posterior DSH, but a significant decrease in CD showed between postoperative and follow-up measurement *p* = 0.05 (Table 2).

**Table 2:** Comparison between anterior DSH, posterior DSH and CD postoperatively and at the follow-up

	Pre-operative	Post-operative	P
Anterior DSH	25.7–63.9	18.6–63.9	0.237 N. S
Range	37.0	34.9	
Mean ± S.D.	11.12	11.20	
Posterior DSH	19.3–66.1	15.1–56.9	0.231 N. S
Range	32.9	30.8	
Mean ± S.D.	11.90	10.85	
Dist. Posterior wall (CD)	1.0–7.7	0.5–5.7	0.05*
Range	3.4	2.9	
Mean ± S.D.	1.51	1.23	

* Significant difference at *p* < 0.05

N.S. Non-significant

No significant correlation was found between GCL and both anterior and posterior DSH (*p* = 0.463/0.401). There was also no significant relationship between radiographic and clinical outcome parameter (Table 3).

**Table 3:** Correlation between GCL, ant. DSH and posterior DSH on both VAS and NDI

	VAS Neck		VAS Arm		ODI	
	Correlation coefficient	*p*-value	Correlation coefficient	*p*-value	Correlation coefficient	*p*-value
Glob. Lordosis	−0.222	0.217	−0.241	0.203	−0.208	0.227
Ant. DSH	−0.017	0.809	−0.113	0.604	−0.012	0.902
Post. DSH	−0.036	0.898	−0.072	0.77	−0.031	0.875

From postoperative to last follow-up, a subsidence of the anterior and posterior DSH and CD were observed of 5.68, 6.38, and 14.71%, respectively.

### Evaluation of the fusion

All the thirty-one patients had radiographic fusion at the last follow-up examination demonstrated by bridging bone between the vertebral bodies or by the absence of motion on dynamic radiographs (Figs. 3 and Fig. 6). After one year postoperatively, one patient suffered from a remarkable neck pain, but nonunion was excluded by CT (Fig. 6).

## Discussion

This study aimed to evaluate radiographic and functional outcome in patients with multilevel degenerative cervical spine disease treated by stand-alone cages for ACCF.

In this series, neither significant subsidence of the cages nor significant loss of lordotic correction were seen. Some patients had dysphagia-related symptoms, but these resided within two days in all patients. Pain and functional outcome improved with surgery and this improvement was maintained during follow-up. Those patients who survived until radiographic follow-up. 31 patients showed a good bony fusion of the surgically addressed segments without any surgical revision or site infection.

Regarding which surgical approach is more effective for the treatment of multilevel CSM, no definitive conclusion could be reached in a systematic review and meta-analysis of Leuo et al. [28]. The anterior approach was associated with better postoperative neurological results compared to the posterior approach. However, there was no apparent difference in the long-term neurological recovery rate.

Patients with multilevel CSM, who underwent ACCF, used to be treated with long anterior strut grafts [29]. Long-term data of patients with one-level corpectomy, cages and additional cervical plate show perfect results with a high fusion rate [30].

However, newer stand-alone cages without an anterior plate may avoid some of the complications seen with conventional methods, especially dysphagia. Dysphagia can range from mild discomfort to inability of control of the muscles used for swallowing. Persistent dysphagia can result in serious medical complications, potential significant morbidity and possible mortality. Although the exact cause of postoperative dysphagia is unknown, it has been speculated that the profile of the plate, adhesions and scar tissue have an impact on the esophagus [29].

Lately in case of ACDF, there is a widespread usage of stand-alone cages in mono- and bi-segmental without anterior plate, which has a lot of problems [31, 32].

Apart from the potential soft-tissue problems that come along with anterior plating of the cervical spine, it may seem obvious that an additional plate adds stability to a multilevel anterior construct from a biomechanical perspective. In biomechanical studies, additional anterior plating provides increased stiffness, especially in extension [33, 34]. On the other hand, it also reverses graft loads and excessively loads the graft or cage in extension, which may promote failure of multilevel constructs [34].

We observed only very little subsidence but no significant loss of lordotic correction. Cage subsidence does not necessarily mean a loss of segmental and GCL. If the collapse of the anterior part of the involved disc space is not higher than that of the posterior part, the local lordosis will be preserved despite the disc space collapse [35, 36]. As surgeons, we should adopt an appropriate technique to avoid over distraction of the disc space, oversizing of the cage and injury of the endplates, especially the anterior border of superior endplate where it is less mineralized [35, 36].

As for the clinical results of the study, subsidence was not found to have any impact on clinical satisfaction.

In a comparable study on cage-assisted interbody fusion with a two-years follow-up, subsidence and cage migration were present in most patients but without clinical sequelae [37]. Furthermore, the two-year radiographic follow-up demonstrated preservation of the physiological alignment of the cervical spine and presence of solid fusion [37].

The limitations of this study include its small sample size. The limited number of patients is not enough to evaluate the percentage of the postoperative dysphagia in such a population [38]. Further studies with long-term follow-up are required to assess the effect of such cages on the adjacent level. Meanwhile, until now, we do not have enough studies about the function and the biomechanics of the expandable stand-alone-cages, which can help us to replace VB with enough extension of the cage to lock the posterior facets and prevent any movement in the segment.

## Conclusion

Application of a stand-alone expandable titanium cage in the cervical spine after one or two level ACCF without additional posterior fixation or anterior plating is a safe procedure that normally results in fusion. In this series, neither significant subsidence of the cages nor significant loss of lordotic correction were seen. However, it may need a surgical skill to limit the over-distraction of the cage to decrease the percentage of the subsidence or to prevent its loosening. In other words, if the cage is primarily stable with a fair and enough distraction forces and there is an intact posterior column, extra anterior plating is not a necessity.

## Supplementary Information

**Additional file 1.** Supplement: Preoperative and Postoperative neurological status.

## Data Availability

You can connect the first author Dr. Mohamed Tohamy or the corresponding author Prof. Christoph Heyde. All data and material are available at any time.

## Abbreviations

ACCF: Anterior cervical corpectomy and fusion
VAS: Visual Analog Scale
NDI: Neck Disability Index
GCL: Global cervical lordosis
FSL: Fusion site lordosis
Ant. DSH: Anterior interbody space height
Pot. DSH: Posterior interbody space height
CD: The distance of the cage to the posterior wall of the vertebral body
ACDF: Anterior cervical discectomy and fusion
SCS: Spinal canal stenosis
VB: Vertebral body
OPLL: Ossification of the posterior longitudinal ligament
CT: Computer tomography
MRI: Magnetic resonance imaging
RA: Rheumatoid arthritis
DM: Diabetes mellitus
