# Supplementary material for: Anterior cervical corpectomy and fusion with stand-alone cages in patients with multilevel degenerative cervical spine disease is safe
Source: BMC Musculoskelet Disord. 2022 Jan 3;23:20. doi: 10.1186/s12891-021-04883-5 (PMC8725343; doi:10.1186/s12891-021-04883-5)
Supplement: Supplementary file 1 — Additional file 1. Supplement: Preoperative and Postoperative neurological status. [file 12891_2021_4883_MOESM1_ESM.docx]

| Patient | Preoperative | Follow-up | Recovery |
| --- | --- | --- | --- |
| 1 | Hyposensitivity C5 bilat. C5-C8 bilat. paresis | Finger flexion both sides incomplete, Otherwise Sensitivity and motoricity intact | Complete |
| 2 | Incomplete sub-C5: upper extremity bilat. and lower extremity bilat. paresis, (ASIA C) | Sensitivity and motoricity intact (ASIA E) | Complete |
| 3 | Incomplete sub-C6: paresis all extremities, bedridden (ASIA C) | Incomplete sub-C6: paresis, mobilized (ASIA D) | Incomplete |
| 4 | Incomplete paraparesis: both legs paresis, hyposensitivity, (ASIA C) | Sensitivity and motoricity intact (ASIA E) | Complete |
| 5 | Hyposensitivity C5–C6 and reduced force C5– C6 on the left | Deltoid left, partial paresis otherwise intact | Incomplete |
| 6 | Tetraparesis sub-C5 | Hyposenstivity all Fingertipps | Incomplete |

Supplement: Preoperative and Postoperative neurological status.
